# Review on the extraction of calcium supplements from eggshells to combat waste generation and chronic calcium deficiency

**DOI:** 10.1007/s11356-021-15158-w

**Published:** 2021-07-16

**Authors:** Akshita Singh, Nachiket Kelkar, Kannan Natarajan, Subbalaxmi Selvaraj

**Affiliations:** Department of Biotechnology, Manipal Institute of Technology, Manipal Academy of Higher Education, Manipal, Karnataka 576104 India

**Keywords:** Calcium supplement, Waste renewal, Eggshell, Calcium extraction, Health

## Abstract

When faced with a plethora of issues, the possibility of one problem becoming the solution of another is a rare, yet beneficial scenario. This report explores the prospect of viewing the accumulation of organic waste matter in India as a potential calcium reservoir to relieve the issue of calcium deficiency in the population. Waste generation has seen gradual growth, and it has created a problem of waste disposal. A large segment of the generated waste primarily consists of food waste which contains significant amounts of nutrients. Food waste such as eggshells, waste from shellfish, bones, and fish scales contain good amounts of bioavailable calcium, and large quantities of this discarded bioavailable calcium remain unused. Global studies show India to have significantly lower levels of calcium intake than the global average, thus increasing the risk of calcium deficiency-related diseases. Furthermore, research shows that for over the past half of the century, the intake of dietary calcium has declined drastically throughout India. This has led to chronic calcium deficiency-related diseases throughout most of the Indian population. Hence, development of calcium supplements from calcium-rich waste material has the potential to not only reduce the strain on waste management, but also to provide the calcium-deficient population with a cheaper alternative to traditional supplements. Owing to the abundance and ease of separation, eggshells have been chosen as the focus of the review. This review highlights and compares their extraction methods of providing cheap calcium supplements while reducing the amount of eggshell waste.

## Introduction

Calcium is one of the most fundamentally critical and essential minerals needed for the human body as the human skeletal system constitutes 98 % calcium (Ross et al. 2011), supplied via dietary sources. It acts as a principal component to the framework of our bodies, and hence, its role is quite discernable. Furthermore, research shows that for over the past half of the century, the intake of dietary calcium has been declining drastically throughout India. Low calcium intake results in a higher occurrence of diseases like beriberi, rickets, and scurvy. As generations progress, the attention towards fulfilling the required calcium intake in humans is rapidly declining, and the consequences of which may lead to lower bone density and many diseases such as osteoporosis and hypocalcemia. Nearly 200 million people worldwide face osteoporosis (Shang and Wu 2019). Osteoporosis causes a steady decrease in bone density (Yedavally-Yellayi et al. 2019). Lower calcium intakes also result in more fragile bones and bone structure deterioration (Arnold et al. 2021). Statistics across several reports indicate a steady downward trend in the amount of calcium obtained via diet (Harinarayan and Akhila 2019). The calcium requirements for the body can be successfully met by dietary intake (Fayet-Moore et al. 2019). Such calcium deficiency figures necessitate the intake of supplements as the optimal solution for this calcium deficiency crisis. Although the current practice of manufacturing these calcium supplements may meet the demand presently, it is an obvious assumption that the assembly, manufacturing, packing, and transportation processes create a number of polluting factors and simultaneously add to global waste. Coincidentally, the solution to this calcium deficiency problem too lies in waste material, with many valuable nutrients in the form of so-called garbage. A report on the in-depth analysis of the waste contents in Mumbai Metropolitan Region revealed that nearly 73% of the garbage was food-related waste. A significant proportion of this food-related waste consists of eggshells, bones, and seafood; all of which hold a high percentage of calcium content. Individuals rarely pay attention to the household waste which is discarded. A high amount of eggshells is dumped in landfills, leading to the production of methane. Still, at the time of need, the scientific community has been exploring methods to manipulate this food-related waste into a viable and beneficial commodity by recycling and repurposing this waste. These conditions call for the development of a calcium supplement that is not only economically feasible but also sustainable and environment-friendly to manufacture. Such low-cost alternatives have also been recommended by the World Health Organization (Bartter et al. 2018). Multiple examples have been cited below, which focus on calcium supplements being sourced from household substances, all of which have a significant amount of calcium traces in them (Nemati et al. 2017; Gaonkar and Chakraborty 2016; Xavier et al. 2013). However, this study chooses to base its scope on calcium supplements derived from eggshells. This report also evaluates and compares appropriate methods of extraction and its feasibility to be transformed into a calcium supplement.

Methods of calcium production and its possible sources have been investigated below, and upon analyzing the distinct procedures between both chemical and biological extraction methods, a clear division is seen when taking environmental factors into account. Relevant methods and techniques used in calcium extraction from eggshells, such as electric discharge-assisted mechanical milling, pulsed electric fields, high energy milling, and high intensity pulsed electric fields, have been studied (Waheed et al. 2019; Barba et al. 2015; Balaz et al. 2013). Furthermore, previous studies with similar sources of calcium and their procedures have been compared and tabulated. Thus, this review attempts to provide an exhaustive overview on calcium supplements that have been created from such waste material and their twofold role as economical alternatives to generic calcium supplements and their contribution in the repurposing and recycling of household waste.

## Issue of waste management in India

### Waste generation in India

**Fig. 1:**
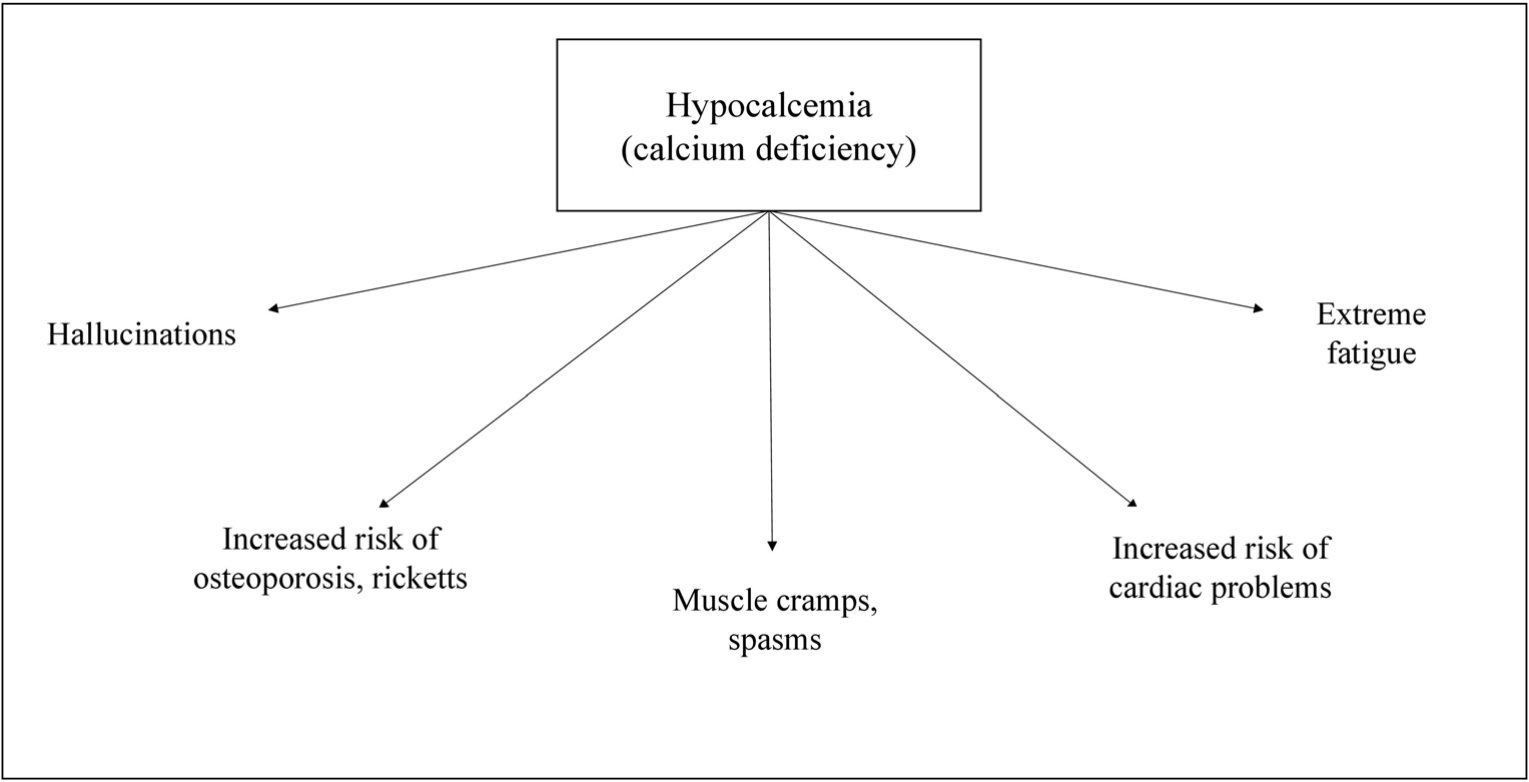
Effects of calcium deficiency in the human body (Harinarayan and Akhila 2019)

India produces nearly 62 million tons of waste per annum, with an average annual growth rate of 4%, from which less than 65% is collected and approximately 15–18% is processed (Balk et al. 2017). The waste generated can be distinguished into three main categories: biomedical (or sanitary and hazardous waste), dry (or recyclable waste), and organic (all biodegradable waste). In a country whose population is 12 times as that of the USA, less than 60% of India’s waste is collected from households, and only 15% of urban waste is processed (Balk et al. 2017). Mumbai Metropolitan Region (MMR) produces per day almost 9,400 metric tons of solid waste, out of which food waste comprised of 73 % whereas plastic is of 3%, as sourced from the environment status report released by the Brihan Mumbai Municipal Corporation (BMC). In India, urban municipal solid waste (MSW) comprises of organics (51 %), recyclables such as paper, plastic, metal, and glass (17.5 %), and inerts (31 %). Urban MSW has 47% moisture content and 7.3 MJ/kg average calorific value. The constituents of MSW in different regions of the India ranges from 50–57 % organics, 28–31 % inerts, and 16–19 % recyclables and between 45 and 51% moisture, and these waste exhibits 6.8–9.8 MJ/kg of calorific value (Cofie et al. 2006; Cooper et al. 2018). Figure 1 depicts the various sources of organic waste in urban populations.

### Calcium content in household waste

Nutrient-wise, the highest losses among nutrients were thiamin, vitamin B12, and vitamin C (130–160 nutrient days/capita/year, respectively). Eighty-eight, 59, and 53 nutrient days/capita/year, respectively, were lost for proteins, dietary energy, and carbohydrates, respectively. Significant losses were reported for under-consumed nutrients in the UK as well: calcium, which was wasted through baked products (27 %), and dairy and poultry (27 %). Primary sources of food folate wastage were observed through fresh greens/vegetables (40 %) and baked products (18 %), as well as dietary fiber (31 and 29%, respectively).

Several dietary studies conducted in the UK show that subgroups of the UK population indicate several concerns regarding the intake of micronutrients such as calcium, iron, vitamin D, dietary fiber, food folate (vitamin B9), and iodine.

### Data for essential foods wasted containing calcium

Under the mentioned food subcategories, it is estimated that the most notable nutrient losses can be attributed to semi-skimmed milk, different types of bread (white, brown, and whole), and different types of cheese. The results indicated wastage of approximately 119 mg/capita/day calcium accounting for 28% of the Reference Nutrient Intake (RNI). The most significant sources are baked products (27% of the total) and dairy/poultry products (27%). Semi-skimmed milk, different types of bread (whole, brown, and white), and hard cheese are estimated to have the highest losses among these food groups.

Spiker et al. (2017) assessed the nutrient losses in the USA for the year 2012 contained 5.9 g dietary fiber, 1.7 μg vitamin D, 33 g protein, 880 mg potassium, 286 mg calcium, and 1,217 kcal energy per capita per day.

The non-inclusion of hospitality or retailer losses led to the expectation that the estimated figures for a household-level food waste study would be markedly lower. The current study proved the claim, calculating 3.4 g fiber, 0.8 μg vitamin D, 10.9 g protein, 486 mg potassium, 120 mg calcium, and 326 kcal energy.

## Addressing the calcium deficiency among populations

### Calcium deficiencies around the world

After an intensive analysis of worldwide calcium deficiency statistics, an interactive global map was developed by Balk et al. (2017), and countries were categorized based on their respective average calcium intakes and changes in intake based on gender, age, and socioeconomic strata. The mean national dietary calcium across 74 countries with sufficient intake ranges from 170 to 1240 mg/day. Several Asian nations show an average intake of around 500 mg/day. Nations across South America and Africa generally exhibited lower than average calcium intake levels spanning from 400 to 700 mg/day. The only countries which exhibited average national calcium intakes that exceeded 1000 mg/day were Northern European countries. For 75% of available nations, survey data was not an accurate measure for the national population sizes. Average calcium intake was observed to be lesser in women as compared to men. Still, there is a lack of correlating statistics across countries regarding relative calcium intake by socioeconomic status, sex, or age. The global calcium intake map revealed that a low average calcium intake is prevalent among many countries.

An overview of the global calcium map shows a significant number of countries having below-average calcium intake, under 400 mg/day. Deficient calcium intake countries are generally situated in Asia and South-East Asian region and include densely populated countries such as India, Indonesia, Vietnam, and China. Nations in the following low intake strata, 400–500 and 500–600 mg/day, were located in South America (Argentina, Bolivia, Brazil) and placed across the Eastern hemisphere and Northern Africa. From 195 countries, only 74 or 38% of countries displayed sufficient data. One hundred twenty-three countries did not possess qualifying survey data on calcium intake.

### Chronic calcium deficiency in India

The daily intake of calcium from dietary sources has been on a steady declining trajectory for India, where statistics have also shown a sharp decline in the g/CU/day calcium content for the last decade. Significant calcium deficiency levels put the Indian population at a higher risk for latency diseases like osteoporosis and osteomalacia (Venkata et al. 2019). The average consumption of calcium in India showed a gradual decrease in the past decade. States such as Maharashtra and Kerala recorded the most deficient calcium consumption statistics in the previous decade. For the combined statistics of Orissa, Gujarat, Kerala, Andhra Pradesh, Tamil Nadu, Karnataka, and Maharashtra, the average consumption between the years of 2011 and 2012 lay between 300 and 400 g/CU/day. The analysis of this data establishes a front on the issue of calcium deficiency in the population (Venkata et al. 2019)

For a Recommended Dietary Allowance (RDA) of calcium, 600 g/CU/day, statistics for various Indian states were compared as distribution percentages of households as a percent of RDA. Some states such as Gujarat, Kerala, and Tamil Nadu showed an increase in percentage households fulfilling RDA of calcium over past decades. However, household percentages fulfilling RDA requirements of calcium dropped in states such as Maharashtra, Madhya Pradesh, and West Bengal severely dropped from the previous decade (2011–2012), with Maharashtra showing the sharpest decline in calcium intake. On average, 37% of households fulfilled calcium RDA requirements in 2011–2012, clearly demonstrating insufficient calcium intake in the population (Venkata et al. 2019).

## Significant sources of calcium in household waste

### Eggshell

Studies on eggshell formation indicate it as an excellent source of calcareous components. Eggshells make up approximately 8–12% of the total weight of the egg (Waheed et al. 2019). The composition of a mineralized eggshell (specifically those of avians) comprises mainly calcium carbonate—96%; the rest of the 4% include minerals like phosphorus, magnesium, and traces of other substances (Hincke et al. 2012). Eggshells have been conclusively proven to have nutritional value (Murakami et al. 2007). However, despite having significant nutritional value and essential components of the human diet, eggshells are considered to have no economic value (Oliveira et al. 2013). Eggshells are thrown away without using any utility they have to offer (Balaz et al. 2021). Multiple studies have shown that eggshells contain significant nutritional value (Waheed et al. 2019; Bartter et al. 2018; Brun et al. 2013). Eggshells contain key factors such as boron and strontium that can help in the prevention of osteoporosis. It is possible to extract almost 40% of the calcium present in eggshells (Lesnierowski and Stangierski 2018). Eggshells exhibit a higher bioavailability in contrast to commercial calcium carbonate (Swiątkiewicz et al. 2015). In spite of having so potential for calcium extraction, eggshells remain an underutilized nutritional source.

#### Global production of eggs

Asia leads in the production of hen eggs, followed by North and South America, Europe, and Africa. Overall, hen egg production has increased from approximately 50 million tons in 2000 to approximately 74–78 million tons in 2018. China accounts for almost 35% of global egg production, with other countries such as the USA, India, Mexico, Brazil, and Japan producing 8%, 7%, 4%, 4%, and 3% of the global eggs, respectively (FAO 2020). There has been a growing trend in egg consumption, with leading consumers being developing countries, as eggs are gradually gaining acceptance as a high-protein food item. Due to this surge in consumption, production has also increased to pacify the demand (Waheed et al. 2020). Globally, egg production has grown almost 1.5 times in the last 3 decades (FAO 2020).

#### Global production of eggshells

The Environmental Protection Agency ranks the problem of eggshell waste as the 15th major food-based pollution source. They can cause various environmental problems such as fungal growth and other problems (Ajala et al. 2018). Industries using eggs in various products are a big contributor of eggshell waste, and these also cause many environmental challenges (Murakami et al. 2007). Most of this eggshell waste which is obtained after consumption goes to waste. Eggshell waste is also sourced from hatcheries. Hence, eggshells are a large and major polluting element (Kingori 2011). This waste is generally dumped in landfills and most landfills are already at full capacity. As eggshell waste is generally protein rich, landfill owners are hesitant to allow eggshell waste, as it attracts rats and other pests. (Waheed et al. 2020) China recorded almost 25 billion kg of eggshell waste, with estimates showing it would reach 35 million metric tons by 2020.

### Shellfish

The composition of calcium in fish and shellfish approximately varies between 15 and 50 mg. Specific to the species of fish, the amount of calcium found in mackerel is less than 15 mg, 15–50 mg in species like catfish, bluefish, oysters, and haddock, and go beyond 50 mg in fish like salmon, trout, and pollock (Xavier et al. 2013)**.**

### Bones

Calcium derived from bone is mainly found in calcium hydroxyapatite (Ca_10_(PO_4_)_6_(OH)_2_) and calcium phosphate (Ca_3_(PO_4_)_2_) structures as a mixture of the two phases. On conducting an elemental analysis of the bones, its mineral composition displayed a high presence of phosphate (56 wt.%) and 31 wt.% of calcium along with other components such as sodium, chloride, magnesium, and potassium (Deydier et al. 2005)**.**

### Fish scales

The source of calcium in fish scales is in the form of “calcium salts,” which cover the surface of collagen present in the tissues. The mineral content of these calcium sources is present in the range of 16–59 wt.% (Alina and Kozlowska 2013).

As the data has demonstrated, there is a significant wastage of food containing a significant amount of bioavailable calcium throughout the world. Furthermore, considering calcium deficiency patterns across the globe, calcium intake is well below the global average (400 mg/day) in India. This factor, coupled with trends demonstrating the decrease in calcium intake levels over time, points out the presence of a significant calcium deficiency in India. From this, it is noted that despite low and further decreasing calcium intakes throughout the world and in India, the bioavailable calcium in household waste, which has the potential of providing nutrition, is not being repurposed. Hence, the production of economically feasible calcium supplements from household waste materials containing bioavailable calcium can serve a twofold purpose. Repurposing of household waste containing bioavailable calcium will help in combating waste generation and supplements produced from this calcium, proposed to be economically feasible, which will help to improve calcium intake levels in areas which demonstrate higher calcium deficiency levels. Hence, this solution will lead to a reduction in waste generation and increase in calcium intake levels; this solution can possess a truly sustainable nature.

The problem with these deficiencies is that the symptoms manifest themselves over time, making it difficult to identify their presence in the general population. When a disease seems to have no immediate cause and effect play at hand, the general awareness is quite low and its symptoms are often exhibited gradually over a longer duration of time, making it difficult to suddenly notice. Due to their neglect towards the required bodily dietary needs, it manifests as a rampant problem throughout the country. Further adding to the problem of calcium deficiency in the body is the decreased amount of vitamin D intake by our bodies. Even if the required dietary intake of calcium is met, the human body still requires a sufficient amount of vitamin D to aid in the absorption of calcium by the body consequently, linking these deficiencies to each other.

As this is a prevalent problem throughout the Indian population, generic supplements for calcium and vitamin D are readily available in the market. Still, they may not necessarily be easily accessible to all sections of society. The lower socioeconomic strata located in rural areas might have rudimentary ways to obtain their micronutrients, for example, using natural resources like the sun (for vitamin D), and farmers harvesting livestock for dairy production. Furthermore, the lower socioeconomic strata could face difficulty with regard to purchasing and continued dosages of expensive calcium supplements (Silva and Nabavi 2019). Unfortunately, the lower socioeconomic strata located in urban areas become the most susceptible to these diseases as their dietary needs often fall short to issues related to finance- and family-related matters, compelling them to sacrifice even the bare minimum.

Developing a calcium supplement synthesized from food-based waste resources is comparatively a more economical and viable option than synthesizing or manufacturing it chemically on a laboratory scale, due to the lack of cost in calcium resources. A calcium supplement manufactured from household waste would not only reduce the environmental strain of decomposition but also alleviate the stress of the most vulnerable groups of society by effectively lowering the cost of the final product, making it readily available for all (Lhamo et al. 2016; Heaney 1991).

## Elementary properties of calcium

An element put in use long before its actual discovery, calcium is one of the most valuable mineral resources for humanity, found abundantly in the earth’s crust. Calcium was first extracted by the electrolysis of mercury oxide and excess lime, which distillation gave pure calcium. Since then, calcium extraction has evolved into more precise and larger-scale productions. The chemical nature of calcium is classified as a solid metal and is designated as the 20th element of the periodic table, placed in S-block (https://www.rsc.org/periodic-table/element/20/calcium). Calcium has a whitish-silvery appearance, which due to its tendency to oxidize, tarnishes rapidly hence protecting against further corrosion. The IUPAC has assigned the standard atomic weight of calcium to be 40.078 Da. The ionic strength of calcium ion in various solutions can be investigated by analyzing the Debye-Huckel equation, which suggests that the charge of an ion affects the influence of ionic strength on the activity coefficient, mainly that as the charge of the ion increases, activity coefficient decreases. In turn, the activity of the calcium ion in biological fluids is a physiological factor that is deemed to be crucial (https://pubchem.ncbi.nlm.nih.gov/compound/Calcium) (Butler 1968).

Other important chemical informations relating to calcium are as follows: the atomic number, 20; atomic mass, 40.08; mass volume at 20 °C, 1.6 g cm^−3^; boiling point, 1484 °C; melting point, 840 °C; Van der Waals radius, 0.197 mm; ionic radius, 0.099 nm; and electronegativity, according to the Pauling scale, is 1.0; it has ten isotopes. The electronic configuration of calcium is [Ar] 4s^2^. The first and second ionization energy are 589.6 kJ mol^−1^ and 1145 kJ mol^−1^, respectively. The standard potential of calcium is −2.87 (Butler 1968). Several isotopes of calcium have been detected and listed, along with their atomic masses and abundance (Table 1). This element comes in several different widely used forms like calcium oxide, calcium sulfate, calcium chloride, and calcium phosphate to name a few, but for the use of supplementation, specifically, calcium carbonate and calcium citrate are of importance.

**Table 1:** Calcium isotopes, respective atomic masses, and abundance (Meija et al. 2016; Coplen et al. 2002; https://ciaaw.org/calcium.htm)

Isotope	Atomic mass (Da)	Isotopic abundance (amount fraction)
^40^Ca	39.962 5909 (2)	0.969 41 (156)
^44^Ca	43.955 482 (2)	0.020 86 (110)
^42^Ca	41.958 618 (1)	0.006 47 (23)
^48^Ca	47.952 5229 (6)	0.001 87 (21)
^43^Ca	42.958 766 (2)	0.001 35 (10)
^46^Ca	45.953 69 (2)	0.00004 (3)

## Availability and abundance of calcium

The bioavailability of calcium is the ratio between potentially absorbable dietary calcium and assimilation of this absorbed calcium into bone (Gueguen and Pointillart 2000). Various dietary components can influence or affect the bioavailability of calcium. Certain food components like vitamin D, lactose, and milk casein phosphopeptides actively promote calcium absorption. Cow milk possesses an excellent bioavailability of calcium (about 30 to 35%). It is approximated that without milk and milk products in the diet, barely 50% of the calcium requirements would be met (Caroli et al. 2011).

Plant-based foods contain many essential components, such as vitamins and minerals, which are vital to a balanced diet and an overall well-being. Plant sources can be excellent sources of calcium. However, plant foods have a certain number of oxalates and phytates, which are inhibitory. They form insoluble calcium salt complexes, thus decreasing calcium absorption (Weaver et al. 1987). Fortified foods are also good sources of bioavailable calcium (Jia et al. 2016). The bioavailability of calcium in certain fortified foods has been compared to milk and milk products, but the calcium content from these foods is not always uniform. Studies on such fortified drinks have shown that the fortifying components settle to the bottom of the carton, and it is not easy to resuspend these components in the beverage (Caroli et al. 2011).

Primary calcium food sources consist of milk-based products, specific vegetables with a low oxalate content, nuts, fortified foodstuffs, and legumes. The most significant quantity of nutrient calcium is sourced from milk and dairy foods. Although the nutritional efficacy of dairy product-based calcium cannot be doubted, some theories or narratives exist as to which source of calcium is biologically better than other sources, such as specific vegetables, salts of calcium, or water with significant mineral content.

In humans, the available calcium present in milk and several milk-derived products such as yoghurts, curd, and cheeses, is less efficiently employed than calcium salts (carbonate, gluconolactate, citramalate, lactate, acetate, and citrate). However, there is a greater rate of absorption than the calcium present in spinach or watercress, as these plants exhibit greater levels of oxalate content, inhibiting intestinal absorption of calcium (Caroli et al. 2011).

## Foods containing high amounts of calcium

The primary source of dietary calcium is from dairy products such as cheese, yoghurt, whey protein, and milk. Leafy greens, tofu and beans like edamame, seeds and nuts such as almonds and amaranth grain, and lentils are an excellent non-dairy source of calcium for the lactose intolerant. Seafood like sardines or canned salmon also contains large amounts of calcium. Vegetables like rhubarb and fruits like fig also contain high levels of calcium. Fortified foods (especially those enriched in calcium) are also a viable option. Apart from these traditional sources, waste material like eggshells, shellfish, bones, and fish scale also possess high amounts of bioavailable calcium.

## Importance of calcium in human and human activity

**Fig. 2:**
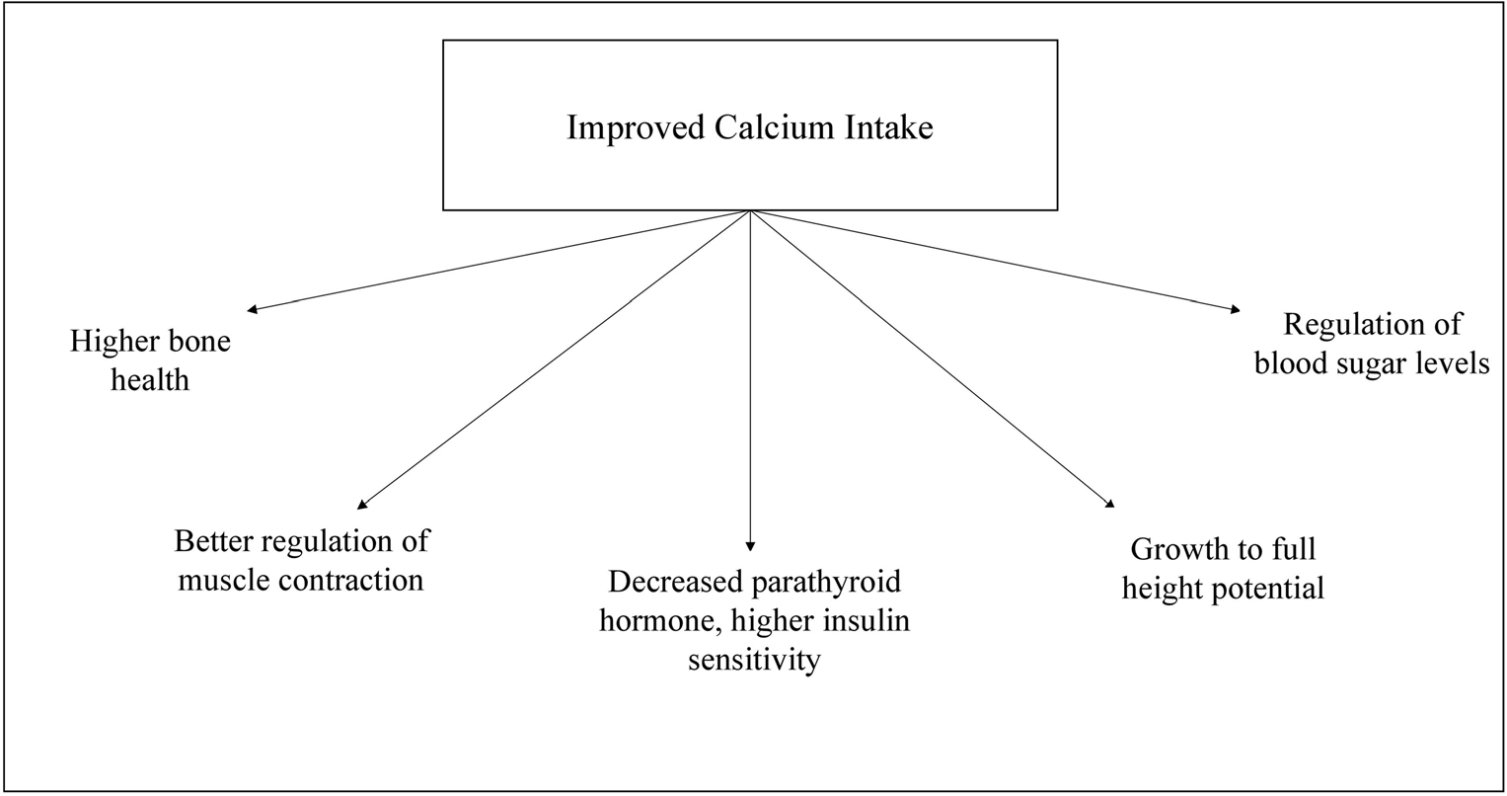
Effects of improved calcium intake (Gueguen and Pointillart 2000)

**Fig. 3:**
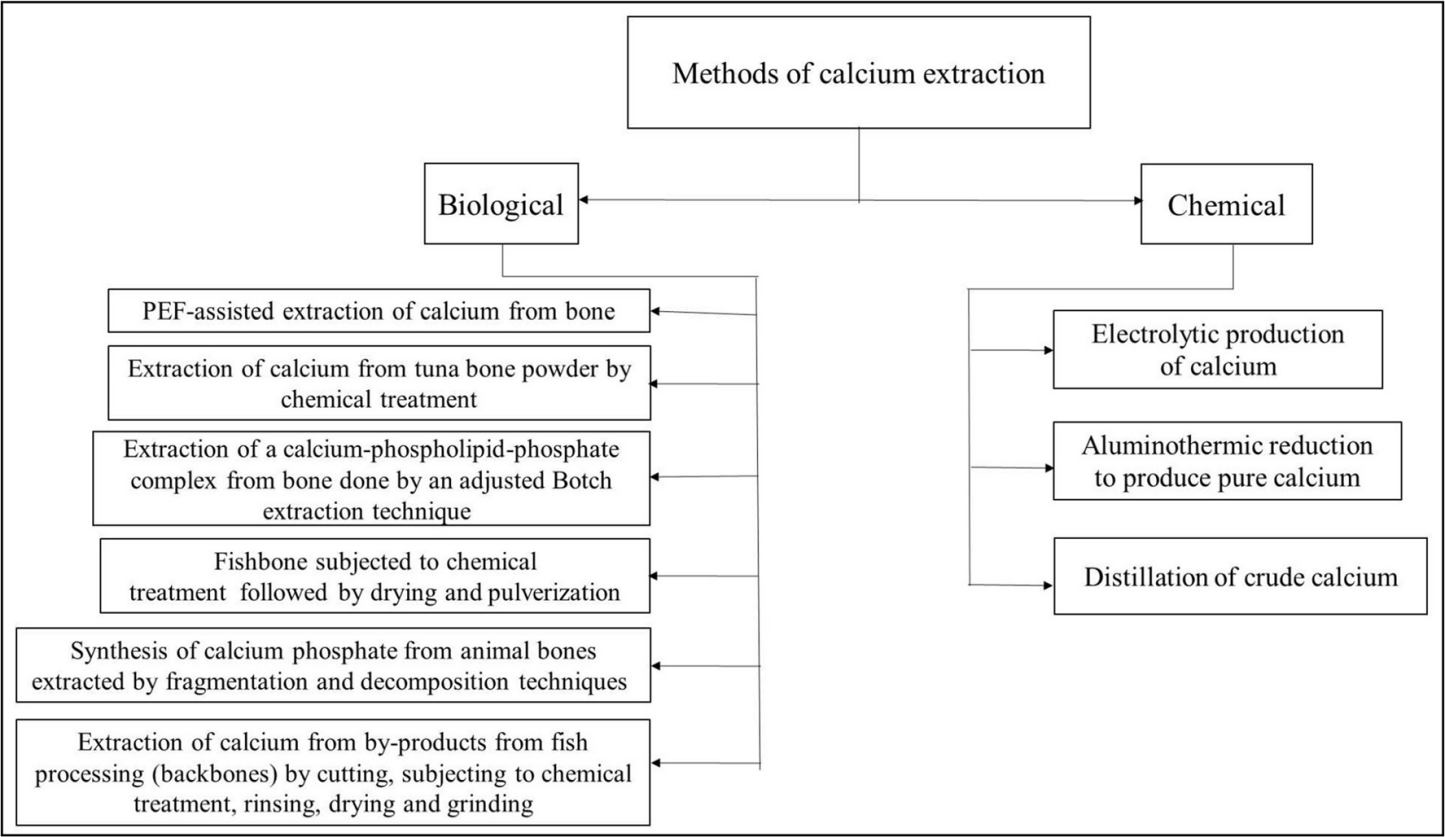
Division of calcium extraction methods into biological and chemical methods

Being the highest mineral content found in our system, the presence of calcium is equally abundant within our bodies as it is on the earth. Calcium ions are essential for carrying out bodily functions such as muscle contraction and relaxation, nerve transmission, hormonal secretion, and intracellular signaling. Vasoconstrictors and vasodilators use calcium by manipulating its levels in the cytosol. The extrinsically derived calcium is stored in the teeth and bones, where it plays a predominantly structural role. The deposition and resorption of calcium into bones is a continuous and essential process called remodeling, necessary for its growth and repair (Harinarayan and Akhila 2019; Gueguen and Pointillart 2000). The consequences due to calcium negligence are depicted in Figure 2; conversely, Figure 3 highlights the benefits of improved calcium intake.

## General methods of calcium extraction

**Fig. 4:**
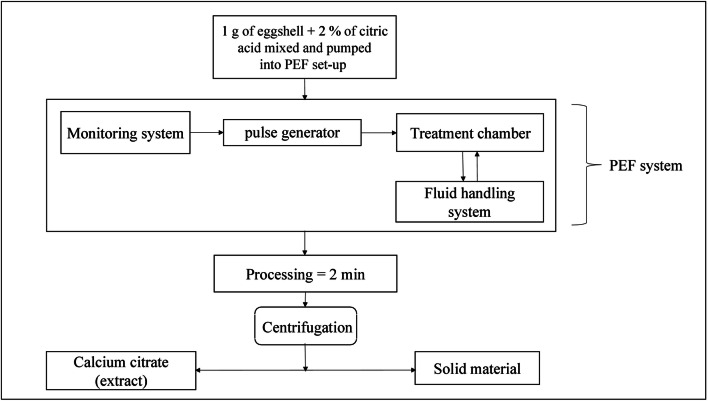
Extraction of calcium using Pulsed electric field **(**PEF) (Waheed et al. 2019)

Extraction of calcium from biological resources involves multiple approaches such as fast and high intensity pulsed electric fields, microfluidic solvent extraction, and alkaline treatment, all collectively termed as biological methods as listed in Figure 4 (Bubel et al. 2016; Boskey and Posner 1976). Many methodologies and other associated downstream processes are involved in its purification. The production of calcium via chemical methods often utilizes procedures that include electrolytic production, distillation, or aluminothermic reduction noted in Figure 4 (Jacob and Srikanth 1990; Wilhelm and Carlson 1964). Either one of the methods is employed based on circumstantial requirements and economic feasibility. The electrolytic production of calcium is generally considered the more contemporary and lucrative option if labor, money, and energy consumption are the criteria for comparison. Processes like distillation or sublimation are taken into consideration when higher percentages of purity are required.

### Calcium extraction methods used for eggshells

Due to the abundance of eggshells in comparison to other waste resources, and their higher calcium content, this study focuses primarily on the extraction of calcium from eggshells.

#### Pulsed electric field (PEF)

**Fig. 5:**
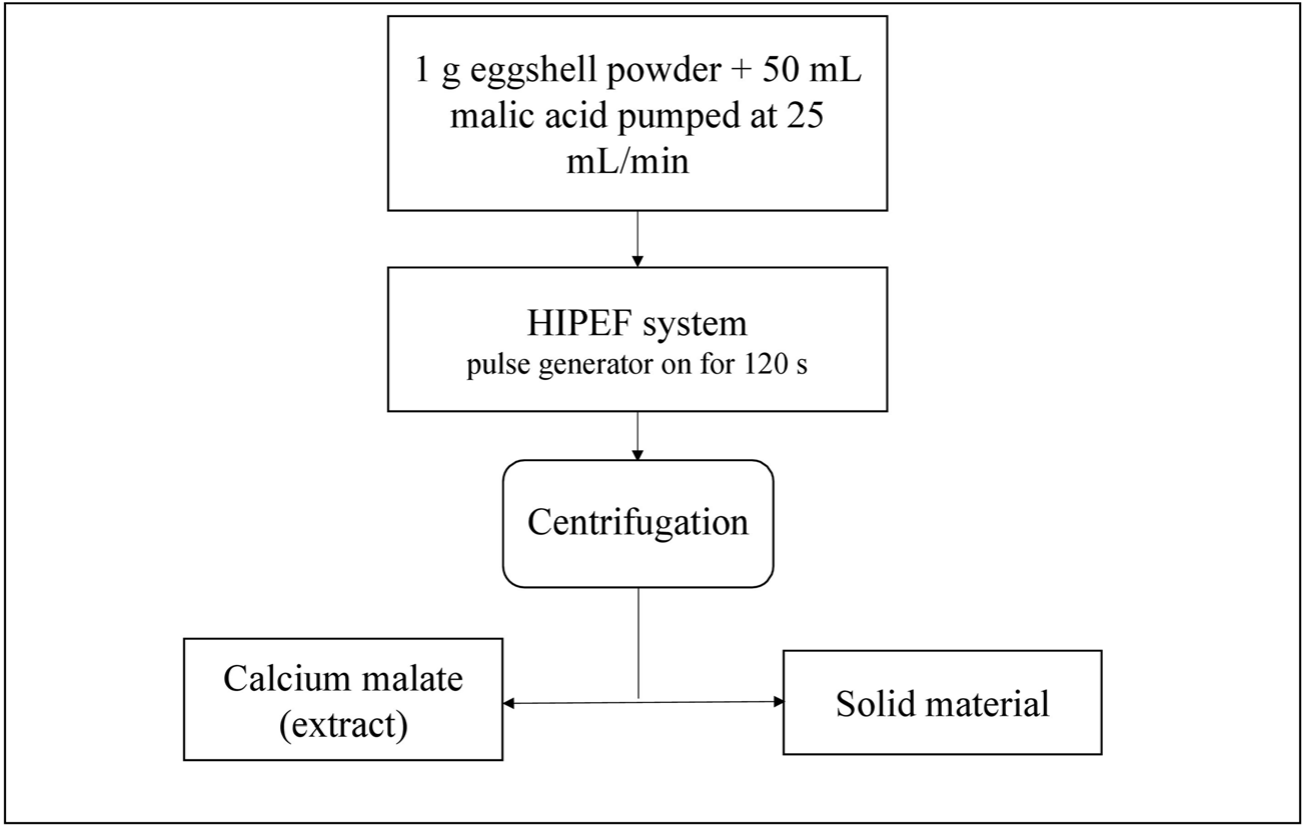
Calcium extraction using High-Intensity Pulsed Electric Fields (HIPEF) (Waheed et al. 2019)

This is a widely used extraction technique employed mainly in the food and pharmaceutical industries. Application of PEF in industries allows for a more sustainable process which is also energy saving. Electroporation is the technique used to bring about the separation of specific materials in PEF. The homogenous proration brought about by PEF is its advantage. The creation of pores subsequently leads to the discharge of cell sap from which cellular contents are extracted. Figure 5 depicts the process of calcium extraction from eggshell using PEF (Waheed et al. 2019; Barba et al. 2015; Yin and He 2008).

#### High-intensity pulsed electric fields (HIPEF)

**Fig. 6:**
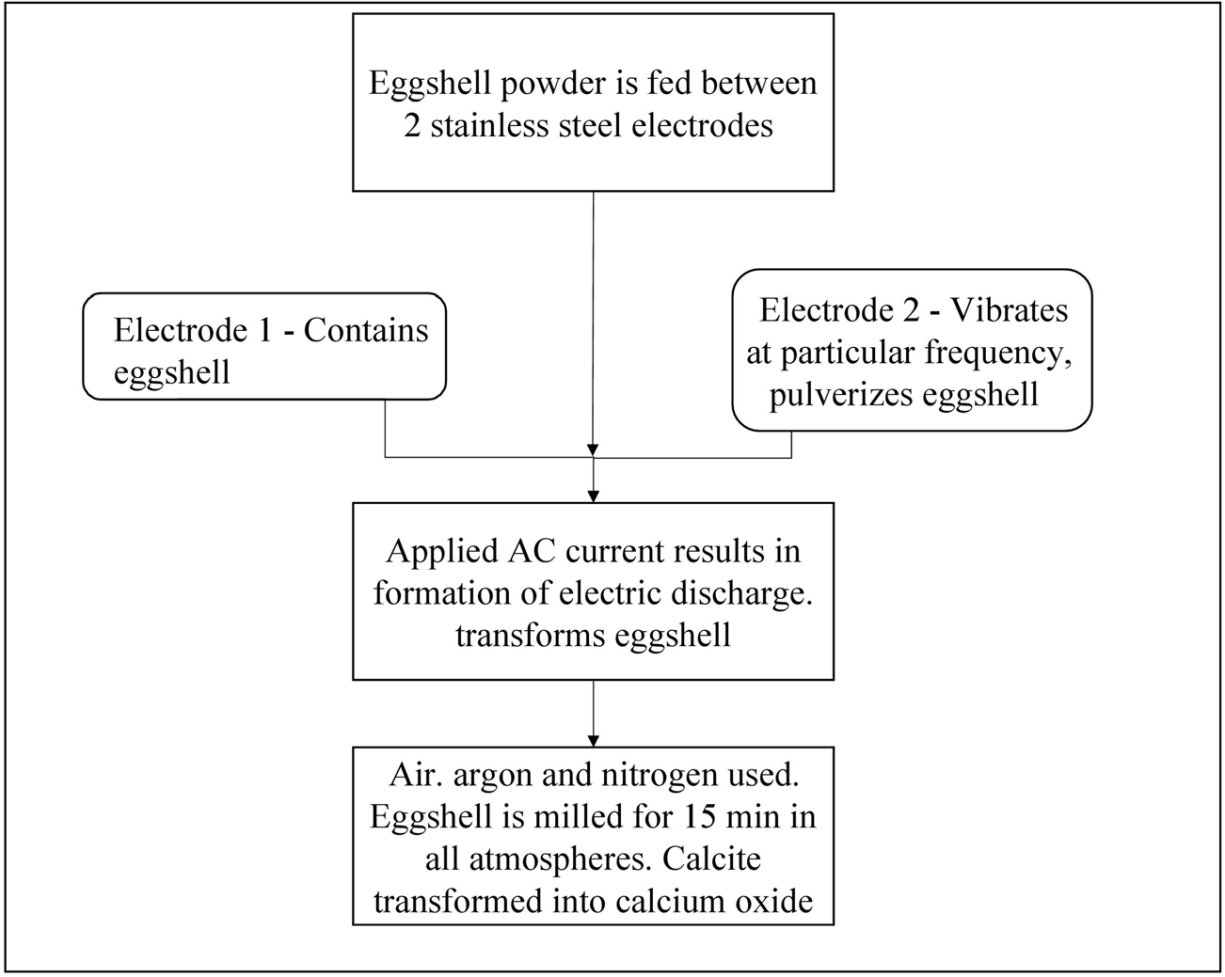
Calcium extraction using Electric discharge assisted mechanical milling (EDAMM) (Waheed et al. 2019)

This method is similar to the PEF technique in terms of supplying high voltage pulses to electrodes placed on either side but instead, delivers a high yield in a short amount of time. The advantages of employing this technique in industrial production are the mild processing temperature required, shorter extraction time, and higher yield; hence, HIPEF is widely used in the pharmaceutical and food industries. Eggshell calcium malate is described as a flowchart in Figure 6 (Yan et al. 2017; Waheed et al. 2019).

#### Electric discharge assisted mechanical milling (EDAMM)

**Fig. 7:**
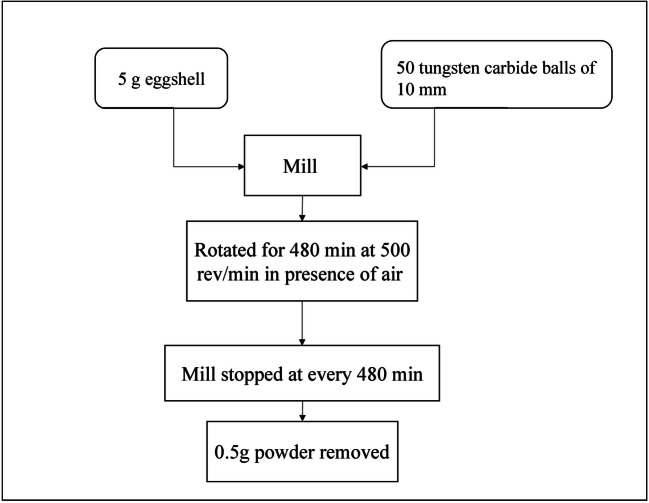
Calcium extraction using High energy milling (HEM) (Waheed et al. 2019)

This technique is used for the synthesis of different high dielectric oxides. This method helps in obtaining highly pure element oxides in a short period of time. Faster reaction is ensured using high voltage impulses and low current levels. Methodology for CaO extraction from eggshell is described in Figure 7 (Balaz et al. 2013).

#### High energy milling (HEM)

Milling processes are used in mineral processing industries and in powder metallurgy where a wide variety of materials are obtained from a pulverized metallic form. Large structures are broken down into smaller ones without allowing clumping. This method is used to create particles for a faster rate of reaction by increasing surface area. However, this process is inefficient and has a high energy requirement. The methodology is shown in Figure 8 (Waheed et al. 2019).

The abundance of eggshells as compared to the other household wastes containing high amounts of bioavailable calcium such as eggshells, shellfish, bones, and fish scales was one of the reasons why the study continues to elaborate on this particular resource. Furthermore, the separation of eggshells from the proteinaceous part, namely egg yolk and albumin, was easy and straight forward. More time-consuming and less efficient separation processes were involved when the separation of muscle and other protein mass from bones and the removal of the proteinaceous part from fish scales and portions of shellfish from the shell were taken into consideration. Also, as demonstrated by Gaonkar and Chakraborty (2016), extraction of CaCO_3_ from oyster shells was shown to be economically and physically expensive. On the other hand, eggshells contained a high amount of CaCO_3_, and the extraction process was comparatively inexpensive, physically feasible, and efficient. The ease and efficiency of calcium extraction from eggshells was noted. Hence, the primary focus of this study centered on methods of calcium extraction based on eggshells as the calcium source.

## A comparison of the eggshell composition of various studies

Eggshells are a great source of calcium as shown in Table 2. Eggshells displayed a good dissolution, which was superior to that of precipitated CaCO_3_. Good dissolution of the chicken eggshell and the presence of other valuable microelements for example, strontium and boron, characterize eggshells and the biomaterial obtained from them as an excellent source for dietary supplements production (Nakano et al. 2019; Łukasz et al. 2015). Eggshell powder has displayed the capacity to improve the bone mineral density and accelerates the remineralization of tooth enamel surfaces. Trace amounts of strontium, minerals, and a lower composition of harmful components are the determining factors that make eggshells the better source of calcium than calcium obtained from oysters.

**Table 2:** The composition of eggshell

Composition	Study 1 (Nasruddeen and Ali 2015)	Study 2 (Hassan 2015)	Study 3 (Schaafsma et al. 2001)
Ash (g/100g)	89.9–91.1	90.2	Not measured
Ca (mg/100g)	35100–35400	35080	40100
Mg (mg/100g)	370–400	262	450
Fe (mg/100g)	Not measured	13.06	2.24
P (mg/100g)	120	150.2	99
Na (mg/100g)	150–170	47.9	Not measured
K (mg/100g)	100–130	50	Not measured
Cu (mg/100g)	Not measured	4.1	0.77
Mn (mg/100g)	Not measured	149.9	Not measured
Sr (μg/100g)	Not measured	Not measured	372
Fl (μg/100g)	Not measured	Not measured	3.75
Se (μg/100g)	Not measured	Not measured	23.5

## Analysis of the various methodologies employed in calcium extraction from eggshells

Biological waste is the most abundant, inexpensive, and exploitable resource that aids in incorporating a sustainable lifestyle within society. Waste under this category largely contributes to environmental degradation by creating pollution but also has the potential to reverse its detrimental effects. Eggshells sourced from poultry prove to be a prime example of the aforementioned resource and also has divergent implementations.

A calcified shell which makes up 10 % of the total egg weight prevents bacterial contamination and comprises of layers that bifurcate into the inner and outer membranes. The functionality of these layers is to retain albumin present in the egg. An ordered crystalline structure of calcium carbonate particles forms this calcite shell. This arrangement done by proteins leads the structure to have multiple minute pores (9000 on average) detracting from its seemingly solid design (Cordeiro and Hincke 2011). The abundance of calcium sources in the shell leads to a plethora of possible applications. The brief methodology on extraction of calcium from eggshells is tabulated in Table 3.

**Table 3:** Methods of calcium extraction from eggshell and their formulations

Sources of eggshells	Procedure	Amount of calcium extracted	References
Fast food industries, poultry farm, houses, bakeries, and hotels	Cooking 12 eggshells in 6 cups of filtered water for 10 min. Then underwent a drying process to achieve a granular and further extracted with 4% (w/v) HCl for 3 h form	0.6 g	Gaonkar and Chakraborty 2016
Different breeds of chicken and other birds found in Bangladesh: Rhode Island red, white Leghorn, domestic duck, and domesticated fowl	A commercially available blending machine was used for processing, sieving, and drying. Then 0.5g of the samples were taken and digested with H_2_O_2_ and ultra-pure HNO_3_. The resulting products transferred for dilution with double distilled water	Overall calcium content found in the eggshells varied from 248 to 386 mg/g	(Islam et al. 2019)
Bakery and white chicken	Researchers analyzed two methods of obtaining a refined eggshell power: mechanical processing, the other using household items such as a rolling pin and sieve to crush and segregate minute particles. Dissolution of the above-obtained products also employed home available resources like vinegar, orange juice, or lemon juice, either of which can also be used.Evaluations were carried out to determine the mineral content of the eggshells. Atomic absorption spectroscopy was used to evaluate the percentage of calcium present	Chicken eggshell powder provided nearly the same results as that of the calcium supplement when calcium absorption was analyzed as a parameter (45.59% ± 14.43% for eggshells and 39.88% ± 16.07% for the supplement)	Brun et al. 2013
Local bakery	Eggshells were subjected to washing, drying, and fragmentation processes. HCl solutions were prepared, to which 100 kg of the processed eggshells was added and stirred. The mixture was subjected to centrifugation and drying; the resulting supernatant was heated, giving rise to CaCl_2_ crystals	These crystals were determined by X-ray diffraction to have the composition of CaCl_2_.2H_2_O; yielding 87.38% (w/w) of eggshell calcium chloride	Garnjanagoonchorn and Changpuak 2007
Egg supplier company Ovopol	For comparison in terms of calcium supplementation, one of the tablets was formulated using calcium citrate extracted from eggshells, and the other derived from synthetic calcium carbonate. This procedure involved roasting the eggshells (for 2 h at 120 °C) with citric acid, followed by tableting. The synthetic calcium carbonate tablet was prepared by forming a granulate followed by drying at 60 °C for 24 h	Calcium content from the calcium citrate extracted from eggshells was 100.0 ± 1.4 mg; and the amount found in the synthetic tablets 100.0 ± 2.7 mg	Barbara et al. 2016

Considerable scientific evidence exists in articles which findings complement the various patents described for the extraction of eggshells (Kingori 2011). Bone mineral density in post-menopausal Dutch women responds positively to the effects of calcium supplementation extracted from eggshells when enhanced with vitamin D and magnesium (Kingori 2011; Schaafsma et al. 2002). Schaafsma et al. 2002 drew this conclusion in the study conducted where the bone density in the hip had a considerable increase for the group supplemented with the calcium extracted from eggshells. Researchers in Japan also identified the effects of eggshell on diseases like osteoporosis, where the combination of vitamin D3 and eggshell powder helped in enhancing bone density and preventing the increase of calcium ions in the blood. Just 2.6–2.8 g of eggshell powder could fulfil the recommended dietary intake quota for an adult (Milbradt et al. 2015).

## Summarizing the findings

The comparative analysis of the many methodologies of calcium extraction from eggshells provides several feasible and economical alternatives for acquiring raw material used in calcium supplementation. Industry scale methodologies such as pulsed energy separation have also been described as a viable approach for eggshells and membrane separation (Yin and He 2008).

Throughout the paper, methodologies of calcium extraction have been discussed, narrowing down to those used for eggshells. The incorporation of statistical evidence on calcium deficiencies in various regions incites clear strategies that can be followed to mitigate the problems of nutrient waste and calcium deficiency in a sustainable manner. A substantial amount of scientific evidence regarding the positive effects of calcium supplementation (Kingori 2011; Schaafsma et al. 2002; Milbradt et al. 2015) was also incorporated to corroborate the nutritional impact of eggshells.

With the methods of eggshell extraction and processing in combination with the well-studied effects of eggshell calcium supplementation, the use of eggshells as a bioresource is propitious. It provides a small yet significant solution to the global waste management issue by becoming an exemplar for reusing biological waste as a solution to modern-day problems.

## Conclusion

In this review, we have looked into the statistics of the waste management issue in India and highlighted the nutritional value lost in the process. Reports about global egg and eggshell production were studied. Thus, a major waste generation and environmental problem was identified due to levels of generation of eggshells and their waste management. (The calcium deficiency in the population and the collected data pertaining to various states were also analyzed in the report.) Calcium contents present in multiple household wastes were mentioned. An in-depth survey of calcium levels across the globe pointed to the fact that calcium deficiency-related diseases have been plaguing all but a few regions. As the benefits of using eggshells outweighed the rest, the study narrowed down on the different extraction methods of eggshells as an attempt to realize its economic viability towards the production of an adequate calcium supplement. Multiple studies, as mentioned, have shown eggshells to possess significant nutritional value which is currently a reservoir of calcium that is not being tapped into. Eggshell waste generation has grown rapidly and will continue to grow further. Due to the menace of overfilled landfills and improper disposal of eggshells, this waste material can have a significant effect on the surrounding as well as contributions to a higher presence of rats and other pestilence. Thus, a twofold solution, which combats not just the issue of waste generation but also one of chronic deficiency, is the need of the hour. This review has tried to correlate the problem of an increasing eggshell waste generation rate, in spite of having significant bioavailable calcium and the fact that while such high amounts of nutritional value calcium go to waste, countries continue to battle chronic calcium deficiencies at alarmingly high rates.

The alarming rate of increase in the generation of household waste with no significant/proportionate effectiveness of any programs aimed at recycling household waste; methods such as the ones stated above is the need of the hour for sustainable waste disposal and recycling. Calcium supplements produced from household waste items, especially eggshells, and their transformation into economically viable models of production can help populations facing calcium deficiencies work towards better nutrition and sustainable waste generation. Thus, in conclusion, this review paper aims to highlight two problems that can be solved by one solution, helping in not only reducing waste that ends up in landfills but by also aiding and improving calcium intake rates throughout the world. As the population increases, consumption follows suit and so does waste generation. We must ensure that as we move forward, not only to reduce our waste generation but also aim to develop repurposing capabilities for waste material that has nutritional value, and ensure that the occurrence of deficiency-related diseases is minimized to a minimal extent.

## Data Availability

The datasets used and/or analyzed during the current study are available from the corresponding author on reasonable request.
